# Mitochondrial ROS-Mediated Metabolic and Cytotoxic Effects of Isoproterenol on Cardiomyocytes Are p53-Dependent and Reversed by Curcumin

**DOI:** 10.3390/molecules27041346

**Published:** 2022-02-16

**Authors:** Jin Hee Lee, Da Hae Kim, MinA Kim, Kyung-Ho Jung, Kyung-Han Lee

**Affiliations:** 1 Department of Nuclear Medicine, Samsung Medical Center, 81 Irwon-ro, Gangnam-gu, Seoul 06351, Korea; mapcar@hanmail.net (J.H.L.); havedh@naver.com (D.H.K.); kmina0730@gmail.com (M.K.); 2 Samsung Advanced Institute for Health and Sciences and Technology, Sungkyunkwan University School of Medicine, Seoul 06351, Korea

**Keywords:** isoproterenol-induced cardiotoxicity, cardiomyocyte, reactive oxygen species, p53, curcumin

## Abstract

Acute β-adrenergic stimulation contributes to heart failure. Here, we investigated the role of p53 in isoproterenol (ISO)-mediated metabolic and oxidative stress effects on cardiomyocytes and explored the direct protective effects offered by the antioxidant nutraceutical curcumin. Differentiated H9C2 rat cardiomyocytes treated with ISO were assayed for glucose uptake, lactate release, and mitochondrial reactive oxygen species (ROS) generation. Survival was assessed by sulforhodamine B assays. Cardiomyocytes showed significantly decreased glucose uptake and lactate release, as well as increased cellular toxicity by ISO treatment. This was accompanied by marked dose-dependent increases of mitochondria-derived ROS. Scavenging with N-acetyl-L-cysteine (NAC) effectively lowered ROS levels, which completely recovered glycolytic metabolism and survival suppressed by ISO. Mechanistically, ISO reduced extracellular-signal-regulated kinase (ERK) activation, whereas it upregulated p53 expression in an ROS-dependent manner. Silencing of p53 with siRNA blocked the ability of ISO to stimulate mitochondrial ROS and suppress glucose uptake, and partially recovered cell survival. Finally, curcumin completely reversed the metabolic and ROS-stimulating effects of ISO. Furthermore, curcumin improved survival of cardiomyocytes exposed to ISO. Thus, ISO suppresses cardiomyocyte glycolytic metabolism and survival by stimulating mitochondrial ROS in a p53-dependent manner. Furthermore, curcumin can efficiently rescue cardiomyocytes from these adverse effects.

## 1. Introduction

Exposure to the β-adrenergic agonist isoproterenol (ISO) is widely used for in vitro and in vivo models to investigate the pathophysiology of cardiomyocyte damage and the beneficial effects of therapeutic agents [1,2].

Mitochondria play a key role in normal cardiac function, as well as in its disturbance [3]. In cardiomyocytes, acute β-adrenergic activity stimulates mitochondrial reactive oxygen species (ROS) production [4,5], which can contribute to heart failure [6]. Mitochondria also fuel the contractile apparatus of myocytes; thus, deficiencies in its energy metabolism contributes to progression of myocardial dysfunction [7,8]. As such, mitochondrial oxidative stress and disrupted glucose metabolism might be a direct mechanism for ISO-induced cardiomyocyte toxicity. A signaling pathway important in the regulation of both glucose metabolism and oxidative stress is the tumor suppresser p53, a key regulator of mitochondria-mediated energy homeostasis and apoptosis [9,10]. In cardiomyocytes, mitochondrial ROS has been suggested to upregulate p53 [5,11]. However, the precise role of p53 in the effects of ISO on cardiomyocyte metabolism, oxidative stress and survival remains unclear.

In respect to cardiomyocyte protection, a beneficial effect against toxic ISO responses might be provided by curcumin, a nutraceutical compound that possesses strong antioxidant activity. Previous studies in living rats demonstrated the capacity of curcumin to reduce ISO-induced myocardial injury, as evidenced by lowered serum myocardial enzymes and myocardial oxidative stress [12,13,14], as well as less histopathologic neutrophil infiltration and myocardial necrosis [12]. However, the pharmacologic effects of ISO and curcumin are influenced in living bodies by a myriad of indirect factors, including hemodynamic, inflammatory, and hormonal responses. Such systemic responses confound interrogations of direct drug effects on target cells. As such, the direct actions of ISO on cardiomyocytes and the protective effects of curcumin are difficult to elucidate using in vivo models.

In the present study, we, therefore, used a cultured cardiomyocyte model to provide a more detailed understanding of the molecular mechanisms underlying ISO-mediated cardiomyocyte toxicity and the protective effects offered by curcumin. We tested the hypothesis that ISO directly stimulates ROS production that suppresses glucose metabolism and survival through p53 signaling, and further investigated the capacity of curcumin to reverse these effects.

## 2. Results

### 2.1. ISO Suppresses H9C2 Cardiomyocyte Glucose Metabolism and Survival

**Figure 1:**
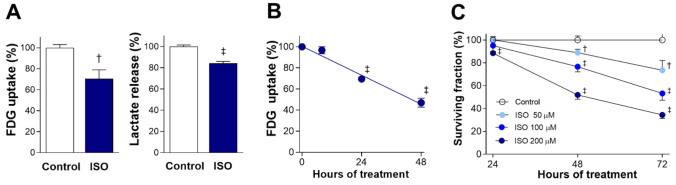
ISO suppresses cardiomyocyte glucose metabolism and survival. (**A**) FDG uptake (**left**) and lactate production (**right**) in differentiated H9C2 cells after 24-h exposure to 200 μM ISO. (**B**) Time course of FDG uptake suppression during exposure to 200 μM ISO. (**C**) Time course of reduced survival over 72-h exposure to graded doses of ISO. Data are the mean ± SE of values obtained from two independent experiments (**A**,**B**; a total of six samples per group) or the mean ± SD of values from a single representative experiment (**C**); four samples per group) relative to that of controls. † *p* < 0.005; ‡ *p* < 0.001 compared with untreated controls.

Exposure of differentiated H9C2 cardiomyocytes to 200 μM ISO for 24 h caused a significant reduction of glucose uptake (as measured by [^18^F]fluoro-2-deoxy-D-glucose (FDG)) to 70.7 ± 8.5% of the untreated level (*p* < 0.005). This was accompanied by a similar reduction of lactate production to 84.0 ± 1.9% of controls (*p* < 0.001; Figure 1A). Time course experiments showed that suppression of glucose uptake by 200 μM ISO began from 24 h and became significantly more pronounced by 48 h, when uptake was reduced to 47.0 ± 4.3% of the control level (*p* < 0.001; Figure 1B).

Sulforhodamine B (SRB) assays demonstrated that ISO dose-dependently and time-dependently suppressed cardiomyocyte survival (Figure 1C). The cytotoxic effect of ISO in doses up to 200 μM was modest at 24 h. At 48 h of exposure, cell survival was reduced to 88.9 ± 3.0% (*p* < 0.005) by 50 μM, 76.6 ± 4.5% (*p* < 0.005) by 100 μM, and 51.9 ± 3.8% of control level by 200 μM of ISO (*p* < 0.001). By 72 h of exposure, cell survival was reduced to 73.6 ± 8.5% (*p* < 0.005), 53.3 ± 6.0% (*p* < 0.001), and 34.4 ± 3.1% of control level (*p* < 0.001) by respective doses.

These results demonstrate that the cytotoxic effect of ISO is preceded by decreases in glucose uptake and lactate production, consistent with suppression of glycolytic metabolism.

### 2.2. ISO Stimulates H9C2 Cardiomyocyte Mitochondrial ROS Generation

**Figure 2:**
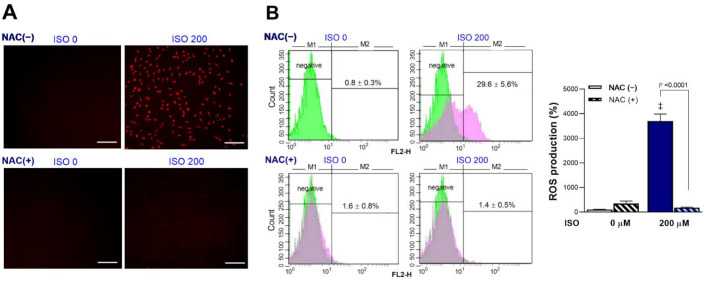
ISO stimulates cardiomyocyte mitochondrial ROS production that is blocked by NAC. (**A**) Representative fluorescent images of differentiated H9C2 cells stained with MitoSOX Red following 24-h exposure to 0 or 200 μM ISO in the absence or presence of 1 mM NAC. Bars indicate 200 μm. (**B**) FACS analysis (**left**) and quantified relative rates of positive cells (**right**) following ISO exposure in the absence or presence of NAC. Bars are the mean ± SE of values obtained from two independent experiments (a total of six samples per group). ‡ *p* < 0.001 compared to vehicle-treated controls.

Exposure of cardiomyocytes to 200 μM ISO for 24 h significantly increased mitochondrial ROS production. Fluorescent microscopy showed that intracellular MitoSOX Red accumulation was increased by ISO, but this was completely blocked by 1 mM N-acetyl-L-cysteine (NAC) (Figure 2A). NAC is an amino acid acylated at the N-terminus with a nucleophilic side chain. Because the free thiol group is readily available for nucleophilic attack, NAC serves as an efficient ROS scavenger that is widely used in experiments to inhibit ROS. FACS analysis confirmed significant increases in the proportion of MitoSOX Red-positive cells from 0.8 ± 0.3% at baseline to 29.6 ± 5.6% after exposure to 200 μM ISO (Figure 2B). This amounted to a 37.0 ± 7.0-fold increase in positive cells by 200 μM ISO (*p* < 0.001). Again, 1 mM of NAC completely blocked the increase of MitoSOX Red-positive cells, which returned to 1.8 ± 0.6-fold that of the control level (Figure 2B). Hence, ISO stimulated a strong increase of cardiomyocyte mitochondrial ROS production.

### 2.3. ROS Scavenging Rescues Cells from ISO-Induced Suppression of Glucose Metabolism and Survival

**Figure 3:**
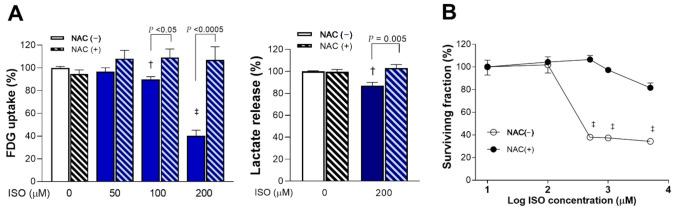
ISO-mediated suppression of glucose metabolism and survival is rescued by ROS scavenging. (**A**) FDG uptake (**left**) and lactate release (**right**) of differentiated H9C2 cells following 24-h exposure to ISO or vehicle in the absence or presence of 1 mM NAC. (**B**) Dose-dependent survival after ISO exposure with or without 1 mM NAC. Data are the mean ± SE of values obtained from two independent experiments (**A**; a total of six samples per group) or the mean ± SD of values from a single representative experiment (**B**; four samples per group) relative to that of controls. † *p* < 0.005; ‡ *p* < 0.001 compared to vehicle-treated controls.

Scavenging of ROS with 1 mM NAC completely reversed ISO-induced suppression of glucose uptake and lactate production. Hence, glucose uptake that was decreased by 100 and 200 μM of ISO to 89.8 ± 2.4% (*p* < 0.005) and 40.3 ± 4.8% of that of controls (*p* < 0.0001) was recovered by NAC to 109.2 ± 7.4% and 107.0 ± 11.6% of controls, respectively (*p* < 0.05 and *p* < 0.0005, compared to absence of NAC; Figure 3A). Similarly, lactate production that was decreased to 87.0 ± 2.9% of that of controls (*p* < 0.005) by 200 μM ISO was recovered to 102.9 ± 3.3% of controls in the presence of NAC (*p* = 0.005, compared to absence of NAC; Figure 3A).

Moreover, 1 mM of NAC completely rescued H9C2 cardiomyocytes from the cytotoxic effect of 24-h exposure to 500 μM ISO that suppressed survival to 37.9 ± 1.5% of that of controls (*p* < 0.001; Figure 3B).

### 2.4. ISO Suppresses ERK Activation and Upregulates p53 Expression in a ROS-Dependent Manner

**Figure 4:**
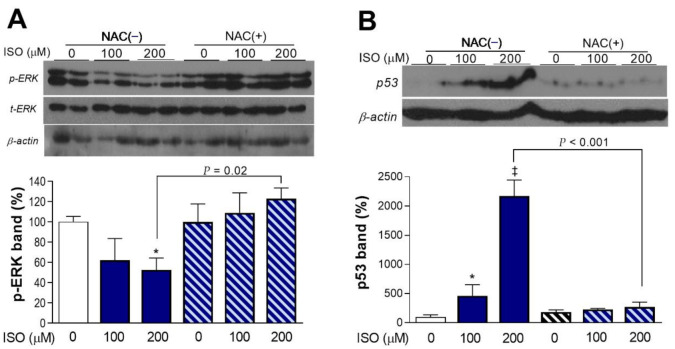
ISO stimulates ERK activation and p53 expression in an ROS-dependent manner. Western blots of activated ERK (*p*-ERK1/2; (**A**)) and p53 (**B**) of differentiated H9C2 cells exposure to 100 or 200 μM ISO for 24 h in the absence or presence of 1 mM NAC. * *p* < 0.05; ‡ *p* < 0.001 compared with untreated controls. Band intensities were normalized by total ERK for *p*-ERK and β-actin for p53. Bars are the mean ± SE of values obtained from two independent experiments (**B**; a total of four samples per group) or the mean ± SD of values from a single representative experiment (**A**; two samples per group) relative to that of controls.

Activation of extracellular-signal-regulated kinase (ERK) signaling, a regulator of cell proliferation in response to stress, was dose-dependently decreased by ISO. Hence, the level of phosphorylated ERK was reduced to 52.4 ± 11.8% of the control level after 24-h exposure to 200 μM ISO. However, there was no change in activated ERK when NAC was present (Figure 4A).

In contrast to ERK activation, expression of p53 was dose-dependently and potently increased by ISO. Hence, there was a substantial 21.7 ± 2.7-fold increase of β-actin protein-corrected p53 level by 200 μM ISO (Figure 4B). This response was also lost in the presence of NAC.

### 2.5. p53 Is Necessary for Full Metabolic and Cytotoxic Activity of ISO

**Figure 5:**
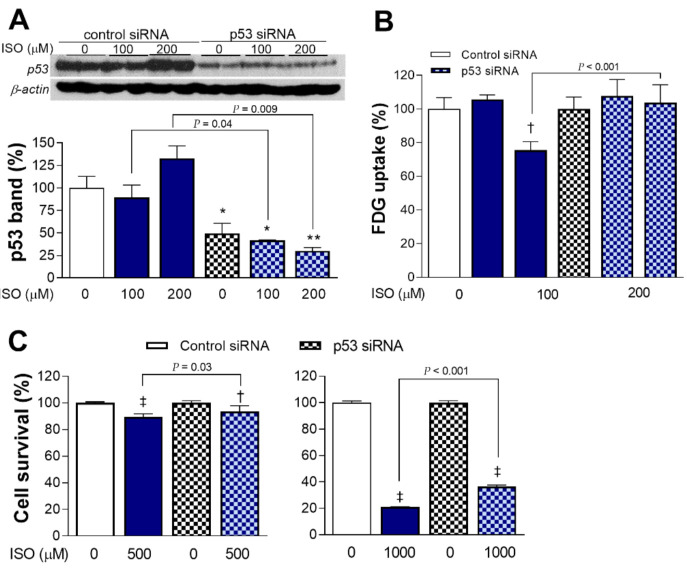
p53 is necessary for full metabolic and cytotoxic actions of ISO. p53 expression (**A**) and FDG uptake (**B**) of differentiated H9C2 cardiomyocytes transfected with p53-specific or scrambled siRNA, followed 24 h later by ISO exposure for 24 h. p53 band intensities were normalized to that of β-actin. Bars are the mean ± SD of values relative to controls obtained from duplicate (**A**) or triplicate (**B**) samples per group. * *p* < 0.05; ** *p* < 0.01; † *p* < 0.005 compared to control siRNA-transfected cells without ISO treatment. (**C**) Survival of H9C2 cells transfected with siRNA and exposed to 500 μM (**left**) and 1 mM (**right**) of ISO as above. Bars are the mean ± SE of data obtained from two independent experiments (a total of eight samples per group) expressed as % relative to that of controls. † *p* < 0.005, ‡ *p* < 0.001 compared to control siRNA-transfected cells without ISO treatment.

Transfection with specific siRNA effectively silenced p53 expression in cardiomyocytes; ISO was no longer able to increase p53 protein accumulation in these cells (Figure 5A). In contrast to a 24.4 ± 5.0% decrease in glucose uptake in control cells transfected with non-target siRNA and exposed to ISO (*p* < 0.001), cells silenced of p53 expression no longer showed reduction in glucose uptake by 200 μM ISO (Figure 5B).

H9C2 cells silenced of p53 expression showed survivals of 93.6 ± 4.2% and 36.5 ± 1.1% after exposure to 0.5 and 1 mM of ISO, respectively, which was improved compared to survivals of 89.5 ± 2.4% (*p* < 0.05) and 21.0 ± 0.4% (*p* < 0.001), respectively, for cells transfected with control siRNA (Figure 5C). This represents a significant reversal of ISO cytotoxicity by p53 silencing.

### 2.6. p53 Is Critical for ISO-Induced Mitochondrial ROS Stimulation

**Figure 6:**
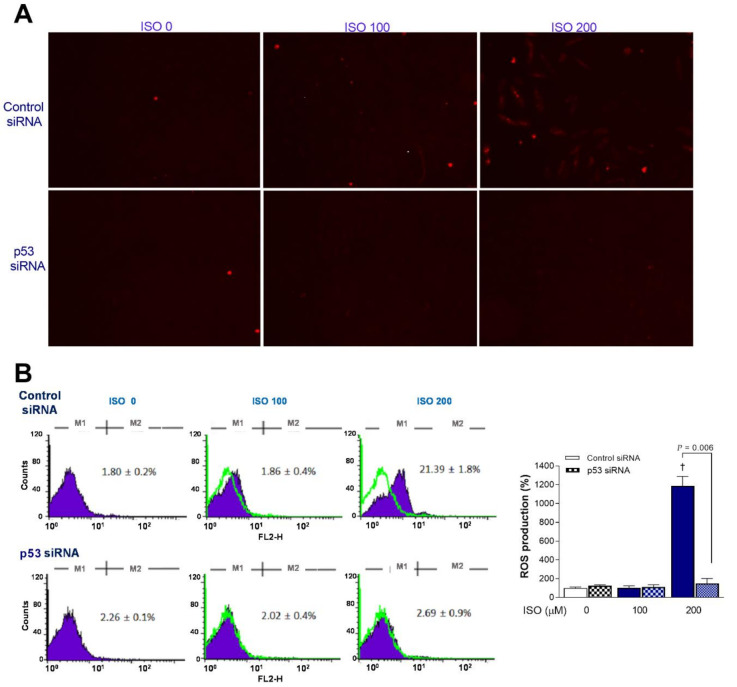
p53 silencing blocks the ability of ISO to stimulate mitochondrial ROS generation. (**A**) Representative MitoSOX Red fluorescent images of H9C2 cardiomyocytes transfected with scrambled (**top**) or p53-specific siRNA (**bottom**) treated 24 h later with ISO for 24 h. (**B**) FACS analysis (**left**) and quantified relative rates of positive cells (**right**) following treatment as above. Bars indicate the mean ± SD of duplicate samples relative to that of controls. † *p* < 0.005 compared to control siRNA-transfected cells without ISO treatment.

The clear increase in mitochondrial ROS based on MitoSOX Red staining by ISO in H9C2 cells transfected with scrambled siRNA was lost in p53-silenced cells (Figure 6A). This was corroborated by FACS analysis, where 200 μM ISO induced a marked 11.9 ± 1.0-fold increase of the proportion of MitoSOX Red-positive cells from 1.9 ± 0.4% to 21.4 ± 3.8% in scrambled siRNA-transfected cells (*p* < 0.001) but did not increase the positivity of p53 siRNA-transfected cells (Figure 6B).

### 2.7. Curcumin Completely Reverses ISO-Stimulated Mitochondrial ROS Production

**Figure 7:**
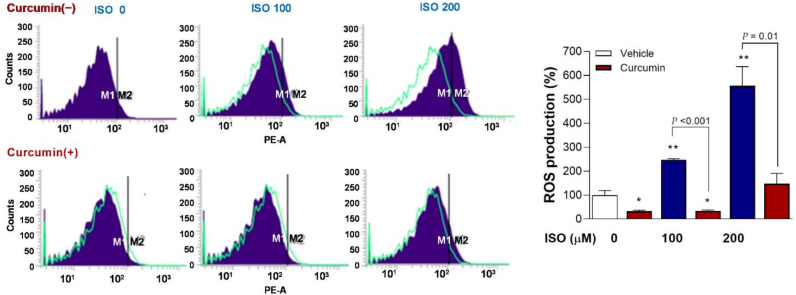
Curcumin completely reverses stimulation of mitochondrial ROS production by ISO. FACS analysis of mitochondrial ROS based on MitoSOX Red in differentiated H9C2 cells (**left**) and quantified relative proportion of positive cells (**right**). Cells were exposed to 0, 100, or 200 μM of ISO for 24 h in the absence or presence of 2.5 µM curcumin. Bars indicate the mean ± SD relative to that of controls obtained from duplicate samples. * *p* < 0.05; ** *p* < 0.01 compared to untreated controls.

FACS analysis of mitochondrial ROS with MitoSOX Red showed that, whereas 100 and 200 μM of ISO caused significant 2.5 ± 0.1-fold (*p* < 0.01) and 5.6 ± 0.8-fold (*p* < 0.01) increases in the proportion of positive-stained H9C2 cells, this ROS stimulating effect was completely reversed in the presence of 2.5 µM curcumin (*p* < 0.001 and < 0.01, compared to cells treated with the same ISO doses without curcumin) (Figure 7).

### 2.8. Curcumin Rescues H9C2 Cardiomyocytes from the Metabolic and Cytotoxic Actions of ISO

**Figure 8:**
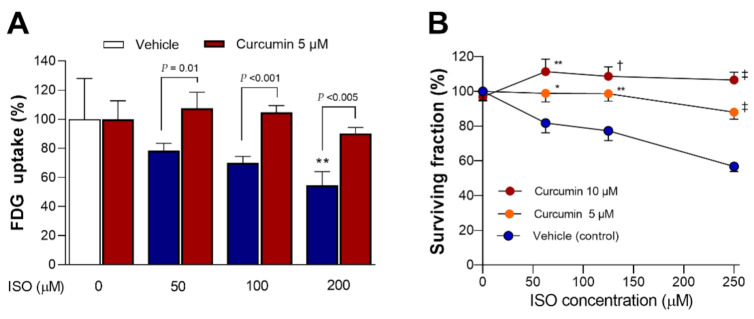
Curcumin protects against ISO-mediated suppression of glucose uptake and survival. FDG uptake at 24 h (**A**) and survival at 48 h (**B**) of differentiated H9C2 cells exposed to ISO in the absence or presence of curcumin. Data are the mean ± SD of triplicate samples (**A**) or the mean ± SE of 10 samples per group from two separate experiments (**B**) expressed as % controls. * *p* < 0.05; ** *p* < 0.01; † *p* < 0.005; ‡ *p* < 0.001 compared to untreated controls (**A**) or cells exposed to the same dose of ISO without curcumin (**B**).

The dose-dependent lowering of glucose uptake by ISO was significantly recovered by curcumin (*p* < 0.01, *p* < 0.001, and *p* < 0.005 compared to cells exposed to 50, 100, and 200 µM ISO without curcumin) (Figure 8A).

When cell survival was tested, curcumin dose-dependently rescued cardiomyocytes from the cytotoxic effects of 48 h exposure to ISO (Figure 8B). Hence, treatment with 5 μM curcumin improved the survival of cells exposed to 125 μM and 250 μM ISO from 77.3 ± 15.9% and 56.7 ± 9.1% to 98.7 ± 13.3% (*p* < 0.01) and 88.1 ± 12.8% (*p* < 0.001) of controls, respectively. Moreover, treatment with 10 μM curcumin further improved survival following respective ISO exposures to 108.7 ± 17.0% (*p* < 0.005) and 106.6 ± 14.3% (*p* < 0.001) of controls, respectively.

## 3. Discussion

This study investigated the direct cellular actions of ISO and the protective effects offered by curcumin using a cultured cardiomyocyte model, free from influences of systemic factors encountered with in vivo models. The results demonstrated that ISO suppressed glycolytic metabolism and survival of H9C2 cells caused by enhanced mitochondrial ROS generation, and further revealed a crucial role of p53 signaling in this response.

Lowered glucose uptake and lactate release by ISO, indicating reduced glycolytic flux, was significant by 24 h, when influence on cell survival was minimal. This demonstrates that the metabolic response of ISO preceded its cytotoxic effect that manifested after 48 h of exposure. At 24 h, the metabolic effect of ISO was accompanied by a marked enhancement of mitochondrial ROS, which can directly and indirectly influence cellular glucose metabolism [15]. In our study, the metabolic response to ISO was blocked completely by ROS scavenging with NAC, confirming dependence on enhanced mitochondrial ROS. Low cardiomyocyte FDG uptake might, thus, serve as a biomarker of ISO-mediated oxidative stress. ROS elevation following ISO exposure led to dose-dependent suppression of cardiomyocyte survival, which, again, was completely recovered by NAC. Thus, both the metabolic and cytotoxic responses to ISO occurred through strong stimulation of mitochondrial ROS production.

Mechanistically, ISO decreased activated ERK level and increased expression of the transcription factor p53 in a manner completely blocked by NAC. Therefore, high mitochondrial ROS increased p53, a master regulator of cellular response to stress. This is consistent with the notion that high ROS can increase p53 accumulation through its stabilization [16]. Branco AF and coworkers also previously showed that ISO exposure increased oxidative stress in H9C2 cardiomyocytes and that this was accompanied by elevated p53 [5]. Our study extends the finding of this previous report by demonstrating that increased p53 in H9C2 cells by ISO is ROS-dependent, and that silencing of p53 expression reverses ISO-mediated decrease of glucose uptake. The substantial increase of p53 may also explain the reduced glycolytic flux observed, which may appear contrary to the notion that mitochondrial dysfunction generally suppresses oxidative respiration and stimulates glycolysis to maintain energy supply. A central role of ROS-induced p53 in the metabolic response of ISO is strongly supported by the complete reversal of decreased glucose uptake by p53 silencing, A previous study reported that p53 signaling can increase myocardial mitochondrial oxygen consumption [17]. This suggests that increased energy produced through oxidative respiration may have contributed to the reduced glycolytic flux we observed. Our results, thus, revealed a key role for p53, a known major player in energy metabolism-modulating programs [9,18], in the metabolic response of ISO.

In addition to induction of p53 by increased mitochondrial ROS, p53, in turn, was necessary for ISO to stimulate mitochondrial ROS production. Indeed, p53 silencing completely abolished the ability of ISO to stimulate mitochondrial ROS production. In previous studies, p53 was similarly required for drug treatment to increase ROS production in renal cells [19] and HeLa cancer cells [20]. In the myocardium, stimulation of mitochondrial oxygen consumption by p53 led to excessive mitochondrial ROS generation [17]. Our data demonstrate that elevated mitochondrial ROS increased p53 accumulation; on the other hand, they also show that p53 was critical for the ability of ISO to stimulate mitochondrial ROS production. Together, these findings point to a positive feedback loop, where mitochondrial ROS upregulates p53 transcription and increased p53 promotes further mitochondrial ROS production. Reciprocal promotive activities between ROS and p53 were previously observed in cancer cells [21]. In our results, the cytotoxic effect of ISO was also lost when p53 expression was silenced. Consistent with its known role for orchestrating growth arrest and programmed cell death [19], p53, thus, exacerbates oxidative stress-induced cytotoxicity in ISO-treated cardiomyocytes.

In our results, ISO also decreased phospho-ERK levels in a fashion that was completely blocked by NAC, indicating a consequence of increased mitochondrial ROS. Although it is known that MAP kinases can induce p53 activation in response to DNA damage, recent findings suggest a more complex picture of functional p53- MAPK interaction. Indeed, activation of phosphatases by p53 suggests a mechanism by which p53 can negatively regulate MAPK signaling. For instance, p53 can induce Pac1 in response to oxidative stress, which in turn is able to inactivate ERK-mediated MAPK signaling [22]. Therefore, the decrease in phospho-ERK observed may be the result of p53-mediated negative regulation. However, clarifying the precise molecular mechanism for this finding was beyond the scope of the present study.

Finally, we explored whether curcumin, a biocompatible polyphenol of the dietary spice turmeric, as the capacity to protect cardiomyocytes from the metabolic and cytotoxic effects of ISO. Previous cardiomyocyte experiments have used nanoparticle preparations for more efficient delivery of curcumin that exhibits low aqueous solubility [23]. Although nanoparticle preparations might have provided greater effects for our own in vitro study, we found that dissolving in DMSO was sufficient to prepare curcumin in the doses required. In our results, curcumin completely recovered cardiomyocyte glucose uptake that was suppressed by ISO. This is consistent with previously observed positive influences of curcumin on glucose uptake in skeletal muscle [24] and myotubes [25]. It is notable that curcumin was able to reduce ROS production in cells without ISO exposure, as well as those exposed to 100 µM ISO, to levels even lower than that of untreated controls. This is consistent with the well-recognized strong antioxidant property of curcumin that occurs both directly by interaction with ROS as well as indirectly through induction of antioxidant proteins [26]. There is accumulating evidence that many of the beneficial effects of curcumin occur by targeting mitochondria [26]. Our group previously revealed that curcumin substantially enhances glucose uptake in cancer cells via suppression of mitochondrial respiration [27]. In the present study, curcumin completely recovered the glucose uptake that was suppressed by ISO in cardiomyocytes. Furthermore, curcumin completely abrogated the capacity of ISO to increase cardiomyocyte mitochondrial ROS production, consistent with the strong antioxidant action of the drug [28]. Importantly, this led to significant and dose-dependent recovery of survival in cardiomyocytes exposed to graded doses of ISO.

## 4. Materials and Methods

### 4.1. Cell Culture and Reagents

H9C2 rat cardiomyoblast cells obtained from the Korean Cell Line Bank (Seoul, Korea) were maintained in a humidified atmosphere at 37 °C and 5% CO_2_ in Dulbecco’s modified Eagle’s medium containing 1 g/L glucose (L-DMEM; Lonza, Switzerland) supplemented with 10% fetal bovine serum (FBS; Serena, Germany) and 1% penicillin/streptomycin. Cells were differentiated over 7 days by incubation in media containing 1% FBS that was changed daily. Experiments were performed 24 to 48 h after seeding of differentiated cells by adding compounds to culture medium. N-Acetyl-L-cysteine (NAC) and curcumin was added 1 h prior to initiation of ISO treatment.

ISO hydrochloride, NAC, and curcumin were from Sigma Aldrich (St. Louis, MO). MitoSOX Red, Lipofectamine RNAi MAX reagent, and GIBCO Opti-MEM media were from Invitrogen Life Technologies (Carlsbad, CA). Antibodies against phosphorylated extracellular-signal-regulated kinase-1/2 (ERK1/2; #9101S), total ERK2 (#9108S) and p53 (#9282S), and anti-rabbit and anti-mouse secondary antibodies were from Cell Signaling Technology (Danvers, MA). Non-targeted small interfering RNA (siRNA), siRNA against p53, and anti-β-actin antibody (#sc-47778) were from Santa Cruz Biotechnology (Dallas, TX, USA).

### 4.2. Sulforhodamine B (SRB) Assay

The surviving cell content was measured with SRB assays on a 96-well plate of 1 × 10^4^ cells per well, as previously described [29]. Briefly, cells were fixed with 10% (*w/v*) trichloroacetic acid at 4 °C for 1 h and stained with 0.4% SRB dye for 30 min; excess dye was removed by washing twice with 1% (*v/v*) acetic acid. Protein-bound dye was dissolved in basic 10 mM Tris-solution and underwent spectrophotometric measurement at 510 nm on a microplate reader.

### 4.3. Glucose Uptake Measurement

Cells seeded in 24-well plates were incubated with 175–370 kBq of [^18^F]-fluorodeoxyglucose (FDG) for 30 min at 37 °C, as previously described [30]. After washing twice with cold phosphate-buffered saline (PBS), cells were lysed with 0.5 N NaOH, and cell-associated radioactivity was measured on a high-energy γ counter (Wallac; Perkin-Elmer, MA, USA). Uptake levels were corrected for cell content by Bradford protein assays.

### 4.4. siRNA Transfection

Gene silencing was performed with siRNA according to manufacturer’s protocol [31]. Briefly, solution-A containing 50 nM of diluted non-targeted siRNA (cell signaling; #6568s) or p53 siRNA (cell signaling; #6231s), and solution-B containing diluted RNAi-MAX transfection reagent were mixed and incubated at room temperature for 20 min. In culture plates, half of the culture medium was removed, the pre-mixed siRNA solution was added, and cells were incubated for 24 h. Culture medium was replaced with fresh complete growth medium before experiments.

### 4.5. Mitochondrial ROS Visualization by Fluorescence Microscopy

Cells in 96-well black plates were grown to 80% confluence, and medium was replaced with fresh phenol red-free RPMI 1640 with 2% FBS. After treatment, medium was replaced with HBSS buffer containing Ca/Mg, 2% BSA and 5 µM of MitoSOX Red mitochondrial superoxide indicator. After 20 min, cells were washed with HBSS buffer containing Ca/Mg but not BSA, which was then added to the cells. Cell fluorescence was visualized and imaged using a TE2000 fluorescence microscopy with 510/590-nm excitation/emission filters (Nikon, Tokyo, Japan).

### 4.6. Mitochondrial ROS Measurement by Flow Cytometry

Cell culture medium was replaced with phenol red-free RPMI 1640 containing 2% FBS; MitoSOX Red was added to a concentration of 5 µM. Following 30 min incubation, cells were washed with PBS, trypsinized, washed with medium and again with HBSS buffer containing Ca/Mg and 1% BSA, and centrifuged. Cells were then resuspended in HBSS buffer, transferred to a sterile strainer tube, and underwent mitochondrial superoxide measurement on a FACSCalibur or FACSVerse flow cytometer (BD Bioscience) using CellQuest or FACSSuite software (Becton-Dickinson, Franklin Lakes, NJ, USA). Data were collected at FSC, SSC, FL2 and FL3 channels.

### 4.7. Western Blot Analysis

Immunoblotting was performed as previously described [32], with 30 µg of cell lysate separated by 12% sodium dodecyl sulfate polyacrylamide gel electrophoresis. Protein transferred to a polyvinylidene difluoride membrane underwent blocking and was incubated overnight at 4 °C with primary antibodies against p53, phospho-ERK1/2, total ERK2, or β-actin (1:1000 dilutions). The membrane was then incubated with secondary antibodies (1:2000) at room temperature for 1 h. Horseradish peroxide (HRP)-linked secondary antibodies were anti-rabbit for p53, phospho-ERK1/2 for total ERK2, and anti-mouse for β-actin. Immunoreactive protein was detected by chemiluminescence; band intensities were quantified on a GS-800 densitometer using Quantity One software (Bio-Rad Laboratories, Hercules, CA, USA).

### 4.8. Statistical Analysis

Data are mean ± SE of values obtained from two independent experiments or mean ± SD of values obtained from a single representative experiment, as specified. Student’s t-tests were used to evaluate significance between groups; *p* < 0.05 was considered statistically significant.

## 5. Conclusions

The β-adrenergic agonist ISO significantly suppressed H9C2 cardiomyocyte glycolytic metabolism and substantially increased mitochondrial ROS production, in a manner that preceded its cytotoxic effect. Mechanistically, ISO upregulated p53, which was necessary for the metabolic response, oxidative stress, and cytotoxic effect of ISO. Importantly, curcumin efficiently rescued cardiomyocytes from the metabolic and mitochondrial ROS-stimulating effects and offered protection from ISO toxicity.

## Data Availability

Data is contained within the article.
